# Human or machine? Do source beliefs shape cognitive bias in post-editing?

**DOI:** 10.3389/fpsyg.2026.1819659

**Published:** 2026-04-22

**Authors:** Jing Cao, Tianli Zhou

**Affiliations:** 1 School of Foreign Languages, Lanzhou Jiaotong University, Lanzhou, Gansu, China; 2 School of Foreign Languages, Zunyi Medical University, Zunyi, Guizhou, China

**Keywords:** cognitive bias, human–AI interaction, labeling effect, post-editing, source beliefs

## Abstract

As artificial intelligence (AI) translation becomes increasingly integrated into professional and educational contexts, perceptions of “machine vs. human” translation may shape translators’ cognitive and evaluative and post-editing behaviors. This study investigates the role of cognitive bias in post-editing, examining how translators’ beliefs about translation sources—human or AI—trigger systematic deviations in evaluation and revision behavior. Sixty master students in Translation and Interpreting participated in a between-subjects experiment: the experimental group received mislabeled texts (human translations labeled as AI, AI translations labeled as human), while the control group was informed of the true sources. Participants evaluated two English-to-Chinese translations on fidelity, fluency, and completeness (5-point Likert scales) and performed post-editing, with modifications recorded for type and rationale. Quantitative analyses (revision counts, quality ratings) and qualitative reflections were examined to explore how cognitive preconceptions modulate linguistic judgment and editing behavior. Findings indicate that perceived AI labels elicited lower trust, increased post-editing intensity, heightened error sensitivity, and more conservative quality ratings, even when translation quality was equivalent. This reveals a subtle form of language-related cognitive bias, in which perceived source identity, rather than objective quality, drives evaluation and modification decisions. By bridging translation studies and cognitive psychology, the study provides empirical insight into human–AI interaction and offers pedagogical implications for fostering critical post-editing literacy and inclusive attitudes toward AI-mediated communication.

## 1 Introduction

The rapid integration of artificial intelligence (AI) translation into professional and educational contexts has fundamentally altered the collaborative dynamics between human translators and automated systems (Leuppert et al., 2025; Yao et al., 2026). As neural machine translation (NMT) systems achieve increasingly competitive output quality, the attributed source of a translation—whether perceived as human-authored or machine-generated—has emerged as a salient contextual variable in professional practice (Béchara et al., 2021). In this shifting landscape, the “label” of the source may carry as much weight as the linguistic properties of the text itself (Asscher and Glikson, 2023). This shift raises a question of both theoretical and practical consequence: to what extent does perceived source identity shape how translators evaluate and engage with translations, independent of the translations’ actual linguistic properties?

Research on cognitive bias in translation has documented systematic deviations from neutral judgment across a range of tasks, including anchoring effects in lexical selection and post-editing (Martikainen and Tiittula, 2016; Litwin, 2025), confirmation bias in quality assessment (Ali, 2025), and the structural influence of evaluator bias on criterion prioritization (Szymczak, 2016). While these studies establish that cognitive bias pervades translation evaluation, they have not examined whether source attribution itself functions as a bias trigger, nor whether its effects differ systematically across evaluative dimensions. The mechanisms through which perceived source identity might distort professional judgment in post-editing thus remain empirically uncharted.

A parallel body of scholarship on source credibility and labeling effects in social cognition offers relevant theoretical grounding. Research consistently demonstrates that source labels can override substantive content in shaping evaluative judgment (Kameda, 1986; Cohen, 2003; Maier et al., 2017). More recently, Leuppert et al. (2025) have shown that AI attribution reduces perceived credibility on interpretive and affective dimensions even when content is held constant, and Greitemeyer et al. (2009) have demonstrated that biased assimilation operates as a function of source position rather than recipient characteristics. Yet this line of research has been conducted primarily with lay audiences in general communication contexts; whether technology-attribution labels produce comparable effects among professional evaluators in translation-specific tasks, and whether individual attitudes toward AI moderate such effects, has not been tested.

To address these gaps, this study draws on Source Credibility Theory (Hovland et al., 1953) as a guiding framework. Hovland et al. (1953) established that perceived source attributes shape evaluative judgment independently of message content, a principle that subsequent research has extended across social, political, and media contexts. Rather than decomposing source credibility into its constituent dimensions of expertise and trustworthiness, the present study reorients this framework toward the categorical activation effects of source labels, investigating how the binary AI versus human attribution functions as a credibility cue that triggers differential evaluative responses prior to and independent of textual engagement. This reorientation reflects the specific conditions of professional post-editing, in which source identity is disclosed as a label rather than inferred from extended interaction, and evaluative responses are shaped by categorical perception rather than deliberative credibility assessment. Three research questions guide the investigation:

(1) Does labeling a translation as AI or human alter evaluative judgments and post-editing behavior?
(2) How do cognitive preconceptions about translation source shape evaluative judgments across fidelity, fluency, and completeness?
(3) How are the variations in AI-related attitudes associated with the magnitude of labeling-induced cognitive bias post-editing behavior?

From a theoretical perspective, this study contributes to the expanding scholarship on cognitive bias in translation by examining source attribution as a schema-activating cue with potential consequences for both evaluation outcomes and the structural weighting of evaluative criteria, dimension of labeling bias that has not yet been systematically examined in previous translation research. It further engages the broader literature on source credibility effects in professional judgment by investigating whether labeling-induced bias operates as a situational rather than dispositional phenomenon. Practically, it holds relevance for the design of translation quality assessment workflows and for the development of pedagogical approaches that cultivate critical post-editing literacy and more calibrated, evidence-based engagement with AI-mediated translation.

As AI translation becomes an increasingly routine feature of professional quality assurance and translator education, the conditions under which this bias may operate are not exceptional but endemic to current practice. This study therefore responds to a pressing need to understand how perceived source identity shapes professional judgment at a moment when human-AI collaboration in translation is rapidly becoming the norm rather than the exception.

## 2 Literature review

### 2.1 Source beliefs and the labeling effect

Source beliefs, defined as recipients’ perceived attributes of communicators, including credibility, expertise, and motivational alignment, play a foundational role in how individuals process and evaluate information. Historically, the study of source-driven judgment traces its origins to the Source Credibility Framework, developed by Hovland et al. (1953). This seminal framework posits that the persuasiveness of a message and its subsequent quality evaluation are heavily mediated by two primary source dimensions: perceived expertise (the source’s ability to provide correct information) and trustworthiness (the source’s motivation to be objective). In the modern digital landscape, these beliefs extend to the “black-box” nature of AI, where the lack of transparency often triggers skepticism (Adadi and Berrada, 2018). Within this paradigm, a source perceived as deficient in these dimensions functions as a “discounting cue,” leading recipients to systematically devalue the message content regardless of its objective merits (Ilić and Damnjanović, 2021).

Early experimental work established that mere labels could shift inferred judgments despite identical substantive content, and that labeling effects are strongest when message diagnosticity is low and prior expectancies serve as base rates in inference processes (Kameda, 1986). This foundational insight has been elaborated across several decades of research in social cognition and persuasion. Cohen (2003) similarly shows that group-level labels can override substantive content in shaping judgment, with source position functioning as a more powerful determinant of evaluative outcome than the properties of the message itself. Greitemeyer et al. (2009) further demonstrate that biased assimilation functions as a consequence of source position rather than recipient characteristics, thereby implying that the situational cue, instead of individual predisposition, constitutes the primary driver of source-attribution effects (Ross and Nisbett, 2011). Building on this situational account, Maier et al. (2017) demonstrate that individuals with strong in-group identification exhibit a belief-consistency effect when processing evaluative content, selectively favoring information congruent with pre-existing schemas while discounting inconsistent evidence. In the context of AI-mediated communication, Bowker and Ciro (2019) emphasize that “machine translation literacy” is becoming a critical determinant of how users perceive and trust AI-mediated output. Extending this line of reasoning, Zhou et al. (2023) extend this understanding by demonstrating that the “fit” between an individual’s decision-making strategy and the task context significantly influences moral and evaluative judgments.

More recently, research has begun to examine how these dynamics operate in the context of human-versus-AI source attribution. At a general level, Yan et al. (2024) discuss the broader “promises and challenges” of generative AI in human learning, suggesting that the perceived authority of AI can either facilitate or hinder objective judgment depending on the user’s prior disposition. Focusing more specifically on credibility evaluation, Leuppert et al. (2025) find that recipients assign lower credibility to AI-authored journalistic content specifically on dimensions associated with interpretive and affective judgment. From the perspective of disclosure design, Morosoli (2025) identifies a “Transparency Dilemma,” in which AI disclosures, although intended to build trust, can paradoxically reduce credibility perceptions depending on the sensitivity of the topic. Extending this argument to memory and misinformation assessment, Mena (2025) adds that the effectiveness of these labels is often contingent on the presence of explanations, which impact how recipients recall the source and assess misinformation credibility.

Historically, much of the foundational evidence regarding professional linguistic judgment has been derived from experimental designs where source information was systematically withheld to ensure an unbiased assessment of the text. For instance, in their comparison of translation architectures, Fischer and Läubli (2020) utilized a blinded setup where the origin of the text (human vs. machine) was not disclosed, ensuring that qualitative perceptions were driven primarily by intrinsic textual properties. Similarly, Toledo-Báez (2024) observed that when participants revised texts without being informed of their origin, their behavioral responses were dictated by objective linguistic characteristics rather than by pre-existing source-based expectations. However, as the presence of AI becomes ubiquitous, researchers have begun to explore how explicit source cues override this objective baseline. Racine (2025), for example, highlights that young adults utilize specific heuristic cues to assess the source credibility of AI-generated content labels, reinforcing the idea that “AI” functions as a distinct, often skeptical, evaluative anchor.

This line of inquiry is still nascent, and its extension to professional translation evaluation contexts in which evaluators possess domain expertise and occupational stakes remains unexplored. Specifically, whether technology-attribution labels, especially when potentially misleading, produce labeling effects comparable in force to social or ideological labels within professional post-editing evaluation and whether such effects operate differentially across distinct evaluative criteria have not been empirically tested.

### 2.2 Cognitive bias in translation

Translation is an inherently bias-susceptible cognitive activity, with multiple systematic patterns of deviation from rational judgment documented across both translation production and quality evaluation (Borg, 2017). Anchoring bias, defined as the tendency to rely excessively on initial information in subsequent decision-making, is among the most extensively documented cognitive biases. Earlier empirical work by Vieira (2014) utilized eye-tracking to demonstrate that cognitive effort is unevenly distributed when translators are anchored by machine output, providing foundational evidence of selective attention. Martikainen and Tiittula (2016) extend this to machine translation post-editing, showing that MT output creates attention distribution effects that shape what editors notice and how they intervene, with anchoring operating through the source material before deliberate analysis begins. Briva-Iglesias et al. (2023) further demonstrate that different post-editing modes (traditional vs. interactive) can shift the user experience and quality perception, potentially introducing new forms of interactive anchoring bias. Litwin (2025) demonstrates that anchoring significantly affects lexical selection and adequacy scores in student translation, showing how initial lexical encounters create cognitive constraints that persist through revision.

Confirmation bias, defined as the tendency to seek and interpret information in ways that confirm preexisting beliefs, has been identified as a particularly consequential mechanism in translation evaluation. Wang (2020) later provided a broader cognitive psychology framework, demonstrating that selective attention to source-salient features is a primary mechanism through which evaluative biases emerge and persist. At the quality assessment level, Szymczak (2016) demonstrates that evaluator biases systematically affect not only scores assigned but the implicit prioritization of criteria during evaluation, pointing to bias operating at the structural level of evaluative logic rather than merely at individual rating outcomes. Munday (2012) identifies critical decision points in translation evaluation where cognitive biases exert disproportionate influence, particularly in situations involving subjective stylistic judgment where no single correct standard exists. This aligns with Asscher and Glikson’s (2023) findings that human evaluations of machine translation are often skewed in ethically or professionally charged situations. Most recently, Ali (2025) documented how these initial source expectations redirect selective attention toward confirming evidence rather than balanced assessment.

The relationship between expertise and bias susceptibility complicates straightforward debiasing proposals. Prassl (2017) finds that professional translators develop more sophisticated cognitive routines but are not immune to bias, exhibiting different but equally entrenched patterns compared to novices, with professional schemas becoming stable rather than flexible over time. Komeili et al. (2019) argue that metacognitive awareness of cognitive bias becomes an ingrained component of professional translation competence as training progresses. Lehka-Paul (2020) demonstrates that individual differences in editing intensity reflect relatively stable decisional styles that persist across task conditions. Furthermore, Yao et al. (2025) argue that measuring a trainee’s effort in AI-assisted post-editing requires a multi-method approach to capture these subtle, ingrained cognitive deviations. A significant gap remains while existing research documents that cognitive biases affect translation evaluation, there is almost no work that maps bias onto specific evaluative dimensions or examines whether source attribution differentially restructures the weight assigned to individual criteria in holistic quality judgment.

### 2.3 Cognitive effort in post-editing

Post-editing machine translation output constitutes a distinct cognitive task in which effort, attention, and revision behavior are jointly shaped by text quality, task demands, and translator characteristics (O’Brien, 2007). In this complex environment, Wickens (2002) Multiple Resources Theory provides a foundational lens for understanding how translators manage the competing cognitive demands of source text analysis, MT verification, and target text production. O’Brien (2006) establishes that pauses during post-editing function as indicators of cognitive load, providing a behavioral metric for understanding how effort fluctuates with task complexity. Alves et al. (2016) provide a granular analysis of how interactive MT environments impact this effort, showing that the “human-in-the-loop” dynamic is not always a linear reduction in load. Moorkens (2018) demonstrates the value of eye-tracking for quantifying cognitive effort in post-editing, showing how behavioral measures can reveal decision-making processes inaccessible to self-report alone. Läubli et al. (2019) find that post-editors working with neural MT output show productivity gains but a slight decrease in textual coherence, suggesting that cognitive efficiency under high-quality MT input may come at the cost of higher-order revision attention. Munková et al. (2021) provide a detailed product and process analysis, showing that the inflectional nature of a language can significantly shift the cognitive effort required during MT post-editing. Ekinci (2023) further demonstrates that specific task interventions, such as error annotation, directly impact the cognitive effort and the final product quality in specialized contexts like subtitle post-editing.

Daems et al. (2017) further identify specific MT error types that significantly elevate post-editing effort, pointing to the interaction between text characteristics and cognitive response. Moving from operational modes to psychological factors, Herbig et al. (2019) propose that efficient translation requires the seamless integration of artificial and human intelligence, yet their work acknowledges that the “human-in-the-loop” process is heavily influenced by how the AI’s contribution is perceived. Vardaro et al. (2019) show that distinct categories of MT errors impose varying levels of cognitive effort, with cognitive load non-uniformly distributed across the post-editing task. Specifically, Béchara et al. (2021) explore how machine translation quality estimation can be integrated into the workflow to assist in effort allocation. In multimodal contexts, Yuan and Wang (2023) highlight the extra visual layer of processing required in complex tasks, which may mirror the cognitive strain of verifying AI-labeled output. Peng et al. (2024) demonstrate that perceived self-efficacy moderates both effort and quality in post-editing, thereby suggesting that psychological states, rather than objective text properties alone, shape the extent to which editors invest cognitive resources. Wang et al. (2024) further demonstrate that AI’s influence is mediated by the user’s “perceived ease of use”, a factor that significantly shapes the actual effort invested. Yao et al. (2025) have recently quantified that AI assistance can transition from a supportive tool to a cognitive distraction, particularly when the user’s focus shifts from content to tool-management. They further provide a cognitive ergonomics perspective, showing how AI assistance can transition from a supportive tool to a cognitive distraction depending on the load (Yao et al., 2026).

Current research also highlights the role of task characteristics in determining productivity and evaluation of algorithms (Castelo et al., 2019). In the context of the translation curriculum, Kenny and Doherty (2014) investigate students’ resistance to statistical machine translation, a precursor to modern AI-related attitudes. Recent studies by Gou (2025) further argue for the collaborative development of human and machine translation from a cognitive approach. Dual-process theory provides a theoretical framework for understanding these interactions (Li and Zhang, 2009): intuitive System 1 processing is more susceptible to biases such as anchoring and confirmation bias, while deliberate System 2 processing is more resistant but more cognitively demanding, with the balance between the two shifting under conditions of cognitive load and time pressure.

A critical gap persists in the current literature, as existing research has not examined whether source attribution systematically alters post-editing revision behavior regardless of actual text quality. The link between a translator’s beliefs about the source and their objective revision counts remains empirically unestablished, and whether individual differences in AI-related beliefs moderate label-induced behavioral change is likewise unexplored.

Despite the extensive body of work discussed above, a significant gap remains regarding the interaction between cognitive bias and professional evaluation. While existing research documents that cognitive biases affect translation evaluation, few studies have mapped these biases onto specific evaluative criteria (Béchara et al., 2021) or examined whether source attribution systematically alters objective post-editing behavior regardless of actual text quality. As noted by Risku et al. (2022), the convergence of sociological and cognitive approaches is essential to fully capture how “praxis” (the act of translating) meets the professional “process” of evaluation. Most importantly, the link between a translator’s internal beliefs and their external revision effort remains empirically unestablished in the context of human-AI interaction. What makes this study unique is its focus on the “labeling effect” as a primary heuristic that can override objective textual properties, restructuring the standards of quality assessment. Through a systematic manipulation of source labels and a multi-dimensional analysis of evaluative scores and revision counts, this research uncovers how perceived identity, rather than objective merit, influences the post-editing behavior. Thus, this study provides a cognitively grounded perspective on the interaction between source-related attitudes and behavioral responses, offering practical insights for refining quality control protocols in professional translation workflows.

## 3 Methodology

### 3.1 Participants

A total of 60 MA students enrolled in a Master of Translation and Interpreting (MTI) program participated in this study on a voluntary basis. Participants were randomly assigned to either an experimental group or a control group, with 30 participants in each group. All participants had received formal training in translation and possessed prior experience with translation revision tasks as part of their MTI coursework. No participant reported prior professional experience in developing or training AI translation systems.

Participants’ ages ranged from 18 to 40 years (*M* = 22.85, *SD* = 3.49), with the majority aged 18–25 (*n* = 51, 85.0%), followed by 26–30 (*n* = 6, 10.0%) and 31–40 (*n* = 3, 5.0%). The sample included students across academic years, with 50%, in their first year, 47% in their second year, and 3% in their third year of the program. This concentration in the first 2 years’ students was due to the fact that these students are in the most intensive stage of their professional translation training, and can serve as a representative cohort. All participants were native speakers of Chinese and used Chinese as their dominant language in academic and professional contexts. Participants consisted of graduate students currently enrolled in a Master of Translation and Interpreting (MTI) program, representing a homogenous group of advanced English users. They shared a robust linguistic foundation characterized by over a decade of formal language education and the successful completion of rigorous academic requirements for entry into professional translation training. This collective background ensures a consistently high level of proficiency in both source-text comprehension and target-language production across the sample. Informed consent was obtained from all participants prior to the experiment.

### 3.2 Instruments

#### 3.2.1 Experimental text

The experimental text was an English expository passage on the topic of Chinese traditional festivals (460 words). This topic was selected on the basis of two criteria: thematic accessibility and cultural specificity. As a subject familiar to Chinese MTI (Master of Translation and Interpretation) students through both lived experience and translation coursework, it minimized the risk of domain-specific knowledge gaps confounding task performance, while its culturally embedded references and descriptive expressions provided sufficient scope for evaluative judgment and revision decisions. The text length was calibrated to allow completion of the full post-editing task within approximately 30 min.

#### 3.2.2 Translation stimuli

Two complete Chinese translations of the source text served as experimental stimuli. One was produced by a professional human translator holding a Level-2 certification from the China Accreditation Test for Translators and Interpreters (CATTI), a nationally recognized qualification examination administered by the Chinese government with approximately 10 years of professional translation experience across academic and commercial domains. The other was generated using ChatGPT (GPT-4, OpenAI). Prior to the experiment, both translations were reviewed by an independent CATTI Level-2 certified translator not otherwise involved in the study, to confirm broad comparability in content coverage and overall quality, with no systematic disparities in fluency or extreme differences in target text length. This review was intended to ensure that observed between-group differences could be attributed to source label perception rather than to objective quality differences between the two translations.

#### 3.2.3 Evaluation form

Translation quality was assessed using a three-dimensional rating scale adapted from the Multidimensional Quality Metrics (MQM) framework (Lommel et al., 2014), evaluating Fidelity, Fluency, and Completeness. Each dimension was rated on a five-point Likert scale (1 = very poor, 5 = excellent). Participants completed the rating scale independently for each translation immediately following the post-editing task, prior to proceeding to the next text.

#### 3.2.4 Post-task questionnaire survey

Following completion of all post-editing and evaluation tasks, participants completed a post-task questionnaire survey comprising four components (see Appendix A for full questionnaire). The first section collected demographic information, including gender, age, English proficiency level, and length of translation study or practice experience. The second component was a matrix item assessing self-reported attitudinal focus during post-editing across four dimensions—language error correction, expressive fluency, cultural appropriateness, and stylistic consistency—rated on a five-point scale (1 = not at all attentive, 5 = extremely attentive). The third component comprised five belief items assessing AI-related attitudes across the dimensions of editing vigilance, quality expectations, trust in human translators, perceptions of occupational replacement, and psychological pressure during AI post-editing, each rated on a five-point Likert scale (1 = strongly disagree, 5 = strongly agree). The fourth component consisted of a comparative perception section in which participants made forced-choice judgments about the two translations regarding overall quality, source attribution, ease of editing, editing confidence, and human stylistic quality, followed by two open-ended questions inviting reflection on experiential differences between the two editing tasks and on the perceived influence of source labels on judgment and behavior.

### 3.3 Research procedures

The study employed a between-subjects design in which participants were randomly assigned to either an experimental group or a control group (*n* = 30 per group). The critical manipulation concerned the source labels displayed alongside the two translation stimuli. In the experimental group, the human-produced translation was labeled “AI translation” and the AI-generated translation was labeled “human translation,” creating a systematic mislabeling condition. In the control group, both translations were presented with their veridical source labels. All other aspects of the procedure, including task instructions, evaluation forms, the questionnaire, and the presentation format, were identical across groups.

The experiment was conducted in a controlled classroom setting with the researcher present throughout. Participants were seated separately to prevent interaction or comparison of responses. The entire session lasted approximately 90 min. Prior to beginning, participants were informed that the study examined translation post-editing and evaluation processes and were instructed to complete all tasks independently, without the use of external reference materials, dictionaries, or translation tools. No practice task was administered prior to the main experiment.

#### 3.3.1 Step 1: first post-editing task

Participants received a printed packet containing the English source text and the first translation stimulus. Crucially, the translation was prominently labeled at the top of the page to establish the purported source: for the Control group, the label reflected the veridical source (e.g., “Human Translation” for Text A); for the Experimental group, a misleading label was applied (e.g., “AI Translation” for the human-produced Text A). To facilitate efficient recording of modifications, both the source text and each translation were pre-numbered at the sentence level, with each sentence assigned a paragraph-and-sentence reference code (e.g., P1S2 for paragraph 1, sentence 2). Participants were not informed at this stage that a second translation of the same source text would follow, and they had no access to the alternative translation.

Participants performed post-editing on the presented translation, recording all revisions on a structured modification analysis sheet. The sheet contained six columns: translation version, source sentence reference number, pre-modification text, post-modification text, modification type, and participant-reported rationale for the modification. This format allowed systematic documentation of each revision while minimizing the recording burden on participants, who were required only to enter the sentence reference code rather than transcribe the full source sentence. The post-editing task was allotted a maximum of 30 min; participants who completed the task earlier were instructed to note their actual completion time on the sheet.

#### 3.3.2 Step 2: quality evaluation of the first translation

Immediately following post-editing, participants completed the translation quality evaluation form for the original translation stimulus—not their revised version. They rated fidelity, fluency, and completeness each on a five-point Likert scale (1 = very poor, 5 = very good). Participants were explicitly instructed that the evaluation targeted the original stimulus as provided, independent of any revisions they had made, and that the purpose was to capture their overall quality judgment of the translation.

Participants took a structured break of approximately 10 min before proceeding to the second round. This interval was intended to reduce carry-over effects from the first editing task on subsequent performance and evaluation.

#### 3.3.3 Step 3: second post-editing task

Following the break, participants received the second translation stimulus. Consistent with the initial manipulation, the labels were again presented according to the participant’s group assignment: the control group received the second text with its correct source label, while the Experimental group received the second text with a deliberate mislabel (e.g., “Human Translation” for the AI-generated Text B). At this stage, the English source text was redistributed and remained available for reference throughout the second task. Participants were thereby aware that both translations corresponded to the same source text. They were instructed, however, to focus exclusively on the translation currently presented and not to refer back to or compare their responses with the previously edited translation. The same post-editing and modification recording procedure as in Step 1 was followed.

#### 3.3.4 Step 4: quality evaluation of the second translation

Upon completing post-editing of the second translation, participants evaluated the original second translation stimulus using the same quality assessment criteria and Likert scales as in Step 2.

#### 3.3.5 Step 5: post-task questionnaire survey

After completing both rounds of post-editing and evaluation, participants completed the post-task questionnaire survey. Administration of the questionnaire was deliberately deferred until after all editing and rating tasks to prevent attitudinal priming effects on participants’ evaluative behavior during the main tasks. The questionnaire took approximately 5 min to complete.

Upon submission of all materials, participants were fully debriefed. They were informed of the true purpose of the study, the nature of the source-label manipulation used in the experimental group, and the actual origins of the two translations. Participants were invited to ask questions and were thanked for their participation.

### 3.4 Data analysis

All quantitative data were processed and analyzed using SPSS (Version 26.0). To isolate the effect of source labeling from text-specific properties, data from both the experimental group (misleading labels) and the control group (authentic labels) were pooled for the primary analysis. A significance threshold of *p* < 0.05 was applied across all inferential tests unless otherwise noted.

To address RQ1, the overall effect of source labeling on evaluative judgment was examined using a paired-samples t-test comparing total quality scores—computed as the sum of scores from the three-dimensional translation quality evaluation form (fidelity, fluency, and completeness; maximum = 15)—across the AI-labeled and Human-labeled conditions, with Cohen’s d reported as the measure of effect size. Prior to analysis, the normality of difference scores for both total quality scores and revision counts was assessed using the Shapiro–Wilk test in SPSS; results indicated that the assumption of normality was sufficiently met in both cases to justify the use of parametric testing. Revision counts, derived from participants’ structured modification analysis sheets, were compared across the two label conditions using a paired-samples *t*-test, with Cohen’s d reported. To examine distributional shifts in evaluation outcomes beyond mean comparisons, total quality scores were categorized into three performance tiers: Excellent (13–15 points), Good (10–12 points), and Pass or Below (≤9 points). This classification was grounded in the score distribution of the Human-labeled condition, where the median score approached 14, with 13 points representing an average rating of above 4 out of 5 across all three dimensions. The proportion of ratings falling within each tier was compared descriptively across label conditions to detect label-induced categorical shifts in evaluative standards. Revision behavior was further examined through two categorical thresholds: high-intensity editing, defined as 10 or more revisions, and minimal editing, defined as 3 or fewer revisions; both were reported as proportions across label conditions. Self-reported editing attention ratings, derived from the matrix item in the post-task questionnaire survey (Q6), were treated as individual exploratory indicators rather than as components of a unified psychometric scale; accordingly, scale-level reliability analysis was not conducted. Mean scores were computed separately for the experimental and control groups across the four attentional dimensions—language error correction, expressive fluency, cultural appropriateness, and stylistic consistency—and group differences were expressed as delta values (*Δ* = Experimental mean minus Control mean) to facilitate direct comparison across dimensions.

To address RQ2, paired-samples t-tests were conducted separately for each dimension of the translation quality evaluation form—fidelity, fluency, and completeness—comparing dimension scores under the AI-labeled and Human-labeled conditions, with Cohen’s *d* reported for each to quantify the magnitude of label-induced suppression per criterion. To examine whether source labeling restructured evaluative priorities beyond direct score suppression, Pearson correlations were computed between each individual dimension score and the total quality score, calculated separately under the AI-labeled and Human-labeled conditions. The resulting correlation coefficients were compared across conditions to assess shifts in the relative diagnostic weight of each evaluative criterion in participants’ holistic quality judgment. A meaningful decrease in a dimension’s correlation with total quality under the AI-labeled condition was interpreted as evidence of reduced evaluative weighting for that criterion.

To address RQ3, the distribution of each AI-related belief item from the post-task questionnaire survey was first characterized descriptively, reporting means, standard deviations, response frequency distributions across the five scale points, and skewness coefficients, to assess the degree of between-person variation available for association analysis. The five belief items were treated as individual exploratory indicators rather than as components of a unified psychometric scale; scale-level reliability analysis was therefore not conducted. Pearson correlations were then computed between each belief item and the revision counts derived from participants’ modification analysis sheets under both the AI-labeled and Human-labeled conditions. The Human-labeled correlations were included as a comparative baseline to assess whether any observed associations were specific to the AI-labeled condition or reflected general editing dispositions independent of label. 95% confidence intervals for all correlations were calculated via Fisher’s (1925) z transformation to provide a conservative basis for interpreting the strength and reliability of observed associations, with confidence intervals overlapping with zero interpreted as indicating a lack of a statistically robust association, regardless of the observed coefficient magnitude.

Qualitative data from the two open-ended items in the post-task questionnaire survey (Q17 and Q18; see Appendix A) were analyzed using keyword frequency analysis. Responses were segmented into individual terms, and high-frequency keywords were identified and tabulated separately for the experimental and control groups.

## 4 Results

### 4.1 The labeling effect on evaluative judgment and post-editing behavior

#### 4.1.1 Source attribution accuracy

Before examining labeling effects, it was necessary to confirm that the experimental manipulation was successful. As shown in Table 1, the mislabeling procedure proved highly effective. In the experimental group, 90.00% of participants identified Text A, which was a human translation bearing an AI label, as machine-produced, while only 3.33% perceived it as reflecting a human translator’s style, providing a valid basis for subsequent between-group comparisons.

**Table 1:** Source attribution accuracy by group (%).

Group	Text	Label	Actual source	Identified as AI (%)	Reflects human style (%)
Experimental	A	AI	Human	90.00	3.33
	B	Human	AI	6.67	93.33
Control	A	Human	Human	10.00	93.33
	B	AI	AI	86.67	6.67

Values not summing to 100 due to the exclusion of “Not Sure” responses.

Conversely, Text B—an AI translation bearing a human label—was recognized as human-like by 93.33% of participants, with only 6.67% correctly attributing it to an AI system. The control group showed correspondingly high attribution accuracy: 86.67% correctly identified the AI-generated text as such, and 93.33% recognized the human translation as reflecting human translator style. These results confirm that the label manipulation was convincingly implemented across both groups, providing a valid basis for.

#### 4.1.2 Effect of source labeling on evaluative judgments

Source credibility theory predicts that source identity cues shape evaluative judgment prior to and independently of message content (Hovland et al., 1953). To assess whether AI versus human labeling produced this effect in translation evaluation, a paired-samples t-test was conducted comparing total quality scores (sum of fidelity, fluency, and completeness; maximum = 15) across the two label conditions. As summarized in Table 2, the mean total quality score for AI-labeled texts (*M* = 12.35, *SD* = 1.48) was significantly lower than for Human-labeled texts (*M* = 3.67, *SD* = 1.35), yielding a mean difference of 1.32 points (*t* = −6.42, *p* < 0.001, *d* = 0.65). This medium-to-large effect size indicates that the AI label functioned as a negative source cue, triggering a systematic devaluation of perceived quality across participants.

**Table 2:** Summary of labeling effects on quality scores and revision counts.

Measure	AI-labeled M (SD)	Human-labeled M (SD)	t	*p*	d
Total quality score (0–15)	12.35 (1.48)	13.67 (1.35)	−6.42	< 0.001***	0.65
Revision count	7.48 (2.96)	6.35 (2.61)	--	0.006**	--

M, mean; SD, standard deviation; d, Cohen’s d. ***p < 0.001.

**Figure 1:**
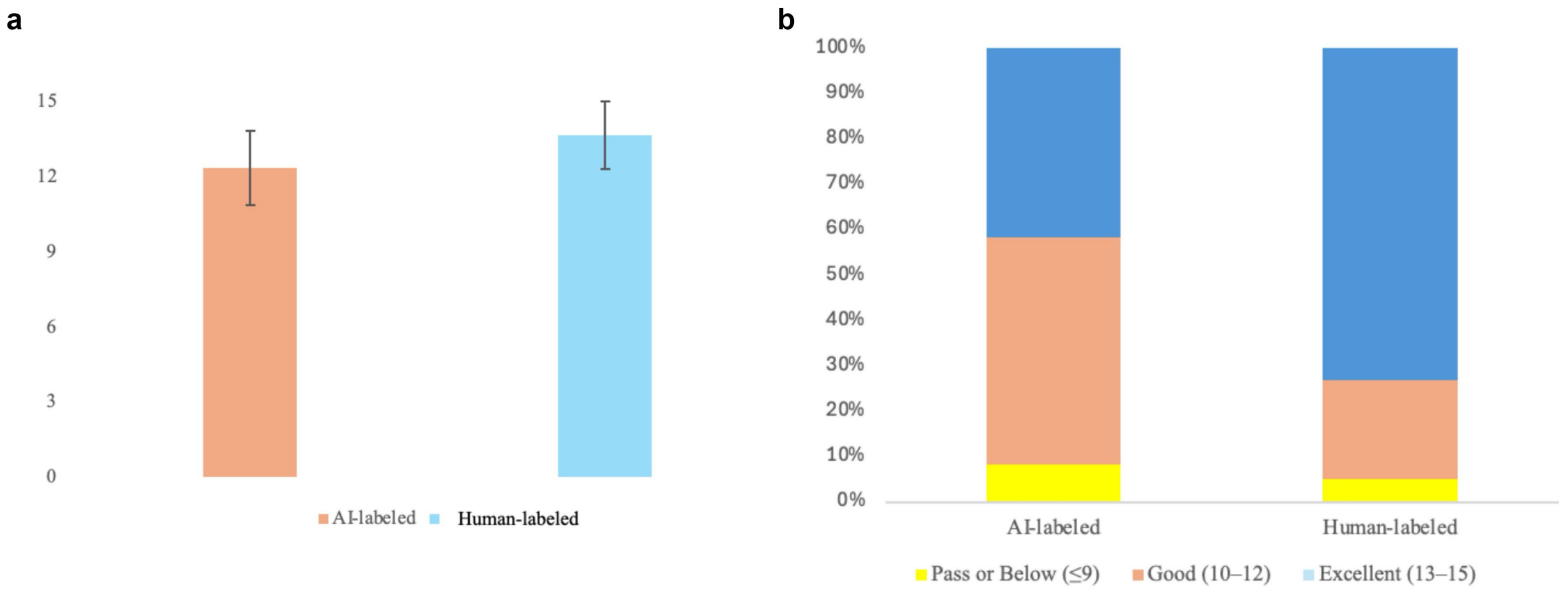
**(a)** Mean total quality scores by label condition (±1 *SD*). **(b)** Distribution of quality rating categories by label condition.

The distributional characteristics of quality ratings further illuminate this pattern of source-driven suppression. As visualized in Figure 1A, the AI-labeled condition (*M* = 12.35, shown in orange) produced not only a lower evaluative mean but also a more dispersed score distribution compared to the Human-labeled condition (*M* = 13.67, shown in blue). This difference in spread is quantified by the higher coefficient of variation in the AI-labeled condition (CV = 12.0%) relative to the Human-labeled condition (CV = 9.9%). The longer error bar in the AI-labeled condition (*SD* = 1.48) compared to the Human-labeled condition (*SD* = 1.35) visually reinforces the finding that source suspicion leads to lower evaluative consensus, as evidenced by the higher coefficient of variation.

A categorical analysis of rating distributions provides a granular view of how this discounting cue restructured participants’ evaluative thresholds. As shown in Figure 1B, under the AI label, the Excellent bracket (13–15 points) plummeted from 73.3 to 41.7%—a sharp decline of 31.6 percentage points. Correspondingly, the Good category (10–12 points) expanded from 21.7 to 50.0% under the AI label, absorbing the ratings displaced from the Excellent tier. The Pass or Below category (≤9 points) remained negligible across both conditions. These structural shifts indicate that the AI label functioned as a “quality ceiling,” systematically preventing evaluators from awarding the highest tier of professional excellence regardless of the texts’ actual properties.

#### 4.1.3 Effect of source labeling on post-editing behavior

Beyond evaluative judgment, source credibility cues have been shown to influence processing effort and behavioral scrutinization (Hovland et al., 1953). Consistent with this account, source labeling in the present study also exerted a significant influence on revision behavior. As previously detailed in Table 2, the AI-labeled condition elicited markedly more editing operations (*M* = 7.48) compared to the Human-labeled condition (*M =* 6.35), representing an increase of approximately 17.8% in revision volume (*p* = 0.006).

**Figure 2:**
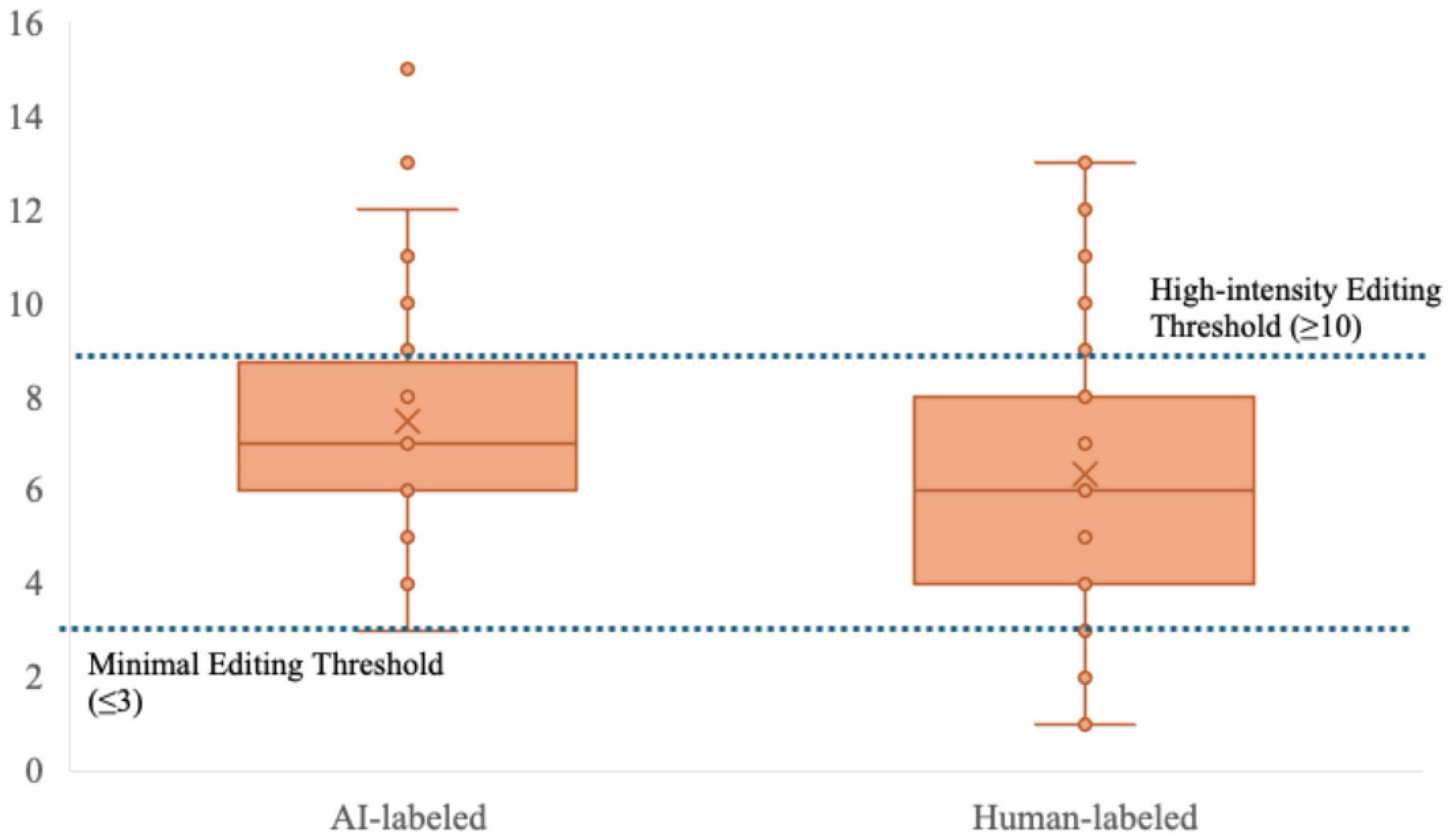
Distribution of revision counts by label condition.

Figure 2 presents the full distribution of revision counts via boxplot, revealing that this increased scrutinization effort operated not only at the level of central tendency but also through systematic shifts in editing intensity thresholds.

In the AI-labeled condition, the upper quartile extended to 10 revisions compared to 8 in the Human-labeled condition, and several high-frequency outliers reached up to 15 revisions, extending the upper tail of the distribution substantially beyond that of the Human-labeled condition. High-intensity editors (≥10 revisions) accounted for 26.7% (n = 16) of the AI group, doubling the 13.3% (*n* = 8) rate found in the Human-labeled baseline. At the lower end of the distribution, minimal revision behavior, defined as 3 or fewer modifications, declined from 11.7% (*n* = 7) in the Human-labeled condition to just 3.3% (*n* = 2) under the AI label.

Complementing these behavioral measures, participants’ retrospective self-ratings of editing attention (Q6 in post-task questionnaire survey) showed a localized decrease in specific evaluative dimensions. While ratings remained consistent across conditions for lower-order concerns—specifically language error correction (Control: *M* = 4.60; Experimental: *M* = 4.57) and expressive fluency (both *M* = 4.57)—the experimental group reported notably lower attention to higher-order textual concerns. Specifically, attention to stylistic consistency was lower in the experimental group (*Δ* = −0.44), as was attention to cultural appropriateness (Δ = −0.14). These self-ratings converge with the behavioral revision data, indicating a shift in the evaluative profile under the AI-labeled condition.

#### 4.1.4 Participants’ reflective accounts

Keyword analysis of open-ended responses in post-task questionnaire survey provided qualitative triangulation for the quantitative patterns identified above. In response to Q17 (how did your experience differ when editing the AI vs. human translation?) the two groups exhibited divergent lexical orientations that corresponded to different levels of textual concern

Participants in the control group (human label) more frequently employed terms associated with higher-order, holistic evaluation, such as *sheng ying* [stiff/unnatural] (*n* = 8), *feng ge* [style] (*n* = 6), *wen hua* [culture] (*n* = 9), and *xi guan* [convention] (*n* = 7). In contrast, the lexical choices of the experimental group (AI label) were predominantly problem-oriented and centered on lower-order detection, with frequent references to *cuo wu* [errors] (*n* = 7), *lian guan* [coherence] (*n* = 4), and *luo ji* [logic] (*n* = 3).

In response to Q18 (Do you think the label affected your judgment or editing behavior?), the groups demonstrated varying degrees of conscious awareness regarding the labeling effect. Among control group participants, while *ying xiang* [influence] was a prominent term (*n* = 22), it frequently co-occurred with the negation *mei you* [did not/no] (*n* = 5). Experimental group participants reported a more uniform recognition of label-driven behavioral shifts, with AI (*n* = 13) and *ying xiang* (*n* = 9) dominating the responses, alongside terms reflecting cognitive bias such as *ke ban* [stereotyped] (*n* = 2) and *yin xiang* [impression] (*n* = 2).

### 4.2 Interaction between cognitive preconceptions and evaluation criteria

Source credibility theory suggests that when a message provides insufficient cues for objective quality assessment, evaluators rely more heavily on source identity as a proxy for quality (Kameda, 1986; Hovland et al., 1953). Source labeling did not suppress translation quality evaluations uniformly. Rather, the three evaluative criteria—Fluency, Fidelity, and Completeness—responded to the AI label with markedly different degrees of sensitivity, and the relative weight each criterion carried in participants’ holistic quality judgments shifted as shown in Table 3.

**Table 3:** Paired-samples *t*-test results by quality dimension (*N* = 60).

Dimension	Human-labeled M (SD)	AI-labeled M (SD)	Diff.	t (59)	*p*	Cohen’s d
Fluency	4.52 (0.62)	3.83 (0.61)	−0.68	−7.30	< 0.001***	−0.94
Fidelity	4.62 (0.58)	4.30 (0.61)	−0.32	−4.32	< 0.001***	−0.56
Completeness	4.52 (0.53)	4.22 (0.55)	−0.30	−3.76	< 0.001***	−0.49

Diff., AI-labeled mean minus Human-labeled mean. Cohen’s d computed from paired differences. ***p < 0.001.

Table 3 presents the paired comparison results. Although all three dimensions yielded statistically significant score reductions under the AI label (all *p*s< 0.001), the magnitude of the effect differed substantially across criteria.

The data in Table 3 show that Fluency—a dimension characterized by lower objective diagnosticity—demonstrated the greatest susceptibility to labeling bias, recording the largest mean reduction (*diff* = −0.68) and a near-large effect size (*d* = −0.94). Fidelity and Completeness, which rely on more explicit source-target comparison (higher diagnosticity), proved comparatively more resilient, yielding moderate effect sizes (*d* = −0.56 and −0.49 respectively).

**Figure 3:**
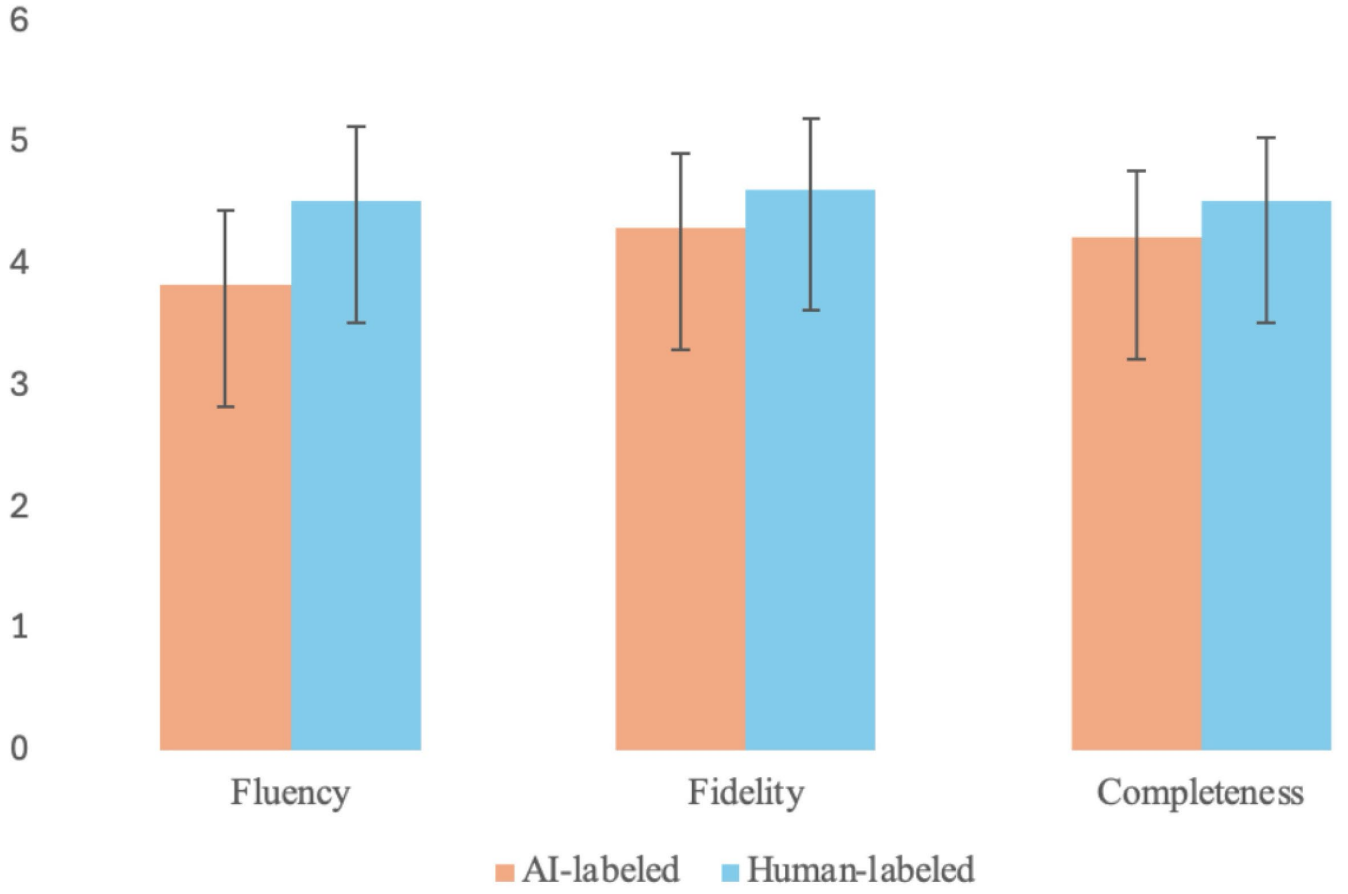
Mean evaluative judgment scores by dimension and label condition.

Figure 3 visualizes the dimensional variance of evaluative judgments through a side-by-side comparison of mean scores. As seen in the chart, the vertical disparity between the human and AI label bars is most striking for Fluency (*M _diff_* = 0.69), creating a pronounced “step-down” effect. This gap progressively constricts as the criteria shift toward Fidelity (*M _diff_* = 0.32) and Completeness (*M _diff_* = 0.30), rendering the gradient of dimensional susceptibility directly visible.

Beyond direct score suppression, source credibility research suggests that source identity cues may alter the implicit criteria through which evaluative judgment is organized, not merely its outcomes (Hovland et al., 1953). Beyond score reductions, Pearson correlations between each dimension and the total quality score were computed separately under each label condition to examine whether source preconceptions alter the evaluative weight carried by each criterion (see Table 4).

**Table 4:** Pearson correlations between dimension scores and total quality score by label condition.

Dimension	r (Human-labeled)	r (AI-labeled)	Change in r	Direction
Fluency	0.791	0.674	−0.117	Decreased
Fidelity	0.779	0.740	−0.039	Stable
Completeness	0.653	0.672	+0.019	Stable

r values reflect Pearson correlation between each dimension score and the summed total quality score (maximum = 15). All coefficients significant at *p* < 0.001.

As summarized in Table 4, the correlational structure between individual dimensions and total quality scores underwent a non-uniform shift. Under the Human-labeled condition, Fluency (*r* =. 791) and Fidelity (*r* = 0.779) contributed almost equally to the holistic judgment. However, under the AI-labeled condition, while Fidelity remained relatively stable, the correlation for Fluency dropped by a magnitude of 0.117, effectively reducing its statistical weight in the overall assessment.

The data reveal a selective devaluation of subjective linguistic dimensions and a concurrent shift in the relative weighting of evaluative criteria, as evidenced by the realignment of Pearson correlations.

**Figure 4:**
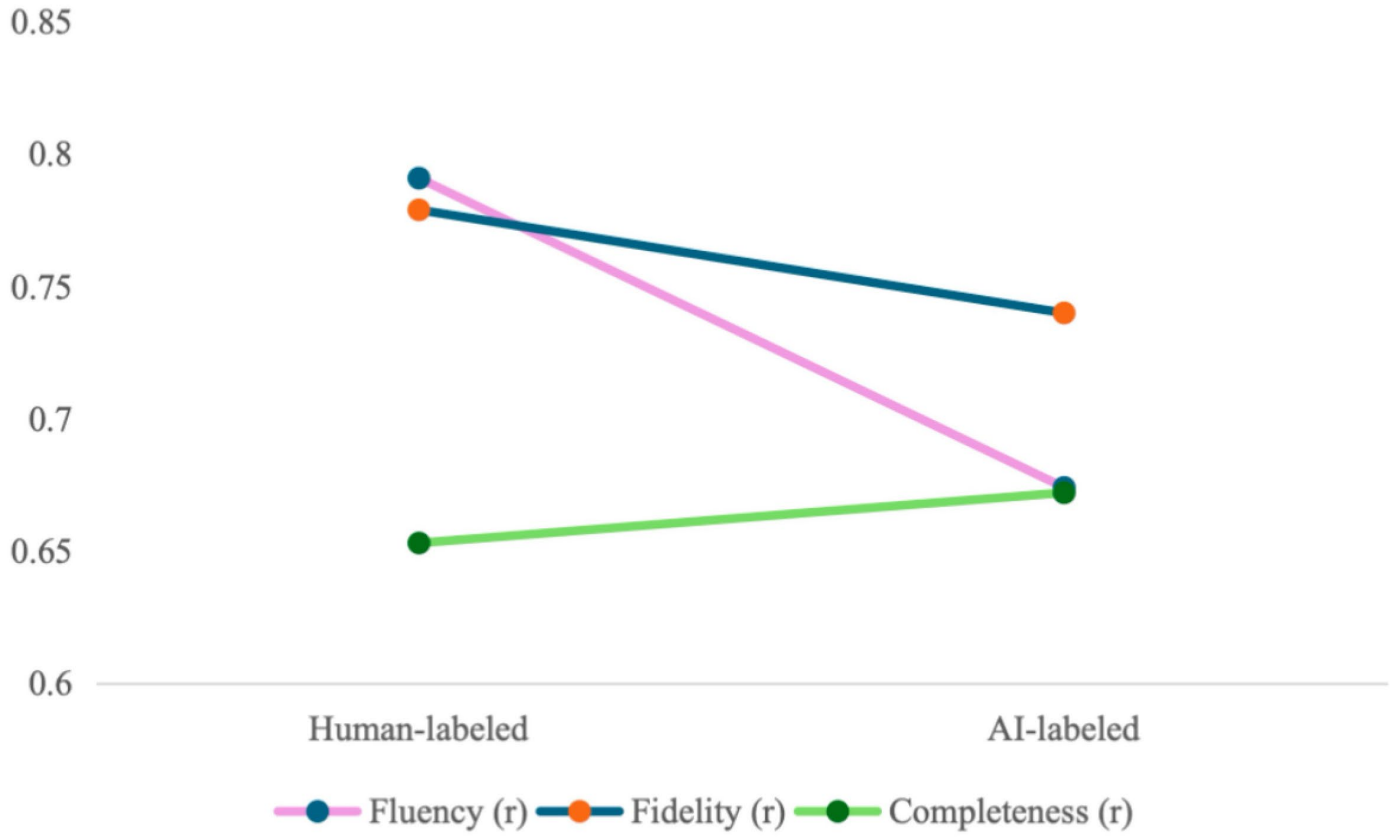
Evaluative weighting shift across dimensions between conditions.

Figure 4 renders this restructuring of evaluative priorities as a dynamic trend. As visualized in the figure, the steep downward trajectory of the Fluency line contrasts sharply with the horizontal stability of Fidelity and Completeness. This divergence results in a distinct crossing point between the Fluency and Fidelity lines as they transition from the human to the AI condition. This intersection provides a direct visual confirmation of the priority reversal identified in the quantitative analysis, where the primary driver of quality judgment switches from stylistic naturalness to semantic accuracy.

### 4.3 Association between AI-related attitudes and labeling-induced bias in post-editing behavior

#### 4.3.1 Distribution of AI-related attitudes

The five attitude items yielded a markedly skewed and concentrated attitudinal profile for most dimensions, as presented in Table 5. Vigilance was the most uniformly held belief—93.3% of participants agreed or strongly agreed that they check more carefully when editing texts they believe to be AI-generated (*M* = 4.47, *SD* = 0.62, skew = −0.74), with no participant selecting ratings 1 or 2. Expectation and Trust similarly clustered at the high end of the scale (*M*s = 4.18 and 4.02, agreement rates 83.3 and 68.3% respectively). By contrast, Replacement and Pressure displayed wider distributional spread across the full 1–5 range, with standard deviations of 1.25 and 0.94 respectively, indicating substantially greater between-person variation on these two dimensions.

**Table 5:** Descriptive distribution of AI-related attitude items (*N* = 60).

Attitude item	1	2	3	4	5	M (SD)	Agree %	Skew
Vigilance: check AI texts more carefully	0	0	6.7	40.0	53.3	4.47 (0.62)	93.3%	−0.74
Expectation: AI quality is improving	0	0	16.7	48.3	35.0	4.18 (0.70)	83.3%	−0.27
Trust: human translators more reliable	0	1.7	30.0	33.3	35.0	4.02 (0.85)	68.3%	−0.20
Replacement: AI will replace some translators	6.7	20.0	20.0	28.3	25.0	3.45 (1.25)	53.3%	−0.34
Pressure: more doubt when editing AI texts	3.3	10.0	53.3	20.0	13.3	3.30 (0.94)	33.3%	0.10

Agree %, combined frequency of ratings 4 and 5. Skew, skewness coefficient. Items are exploratory indicators and do not constitute a validated psychometric scale.

#### 4.3.2 Associations between AI-related attitudes and post-editing revision counts

Table 5 presents the Pearson correlations between each attitude item and revision counts under both the AI-labeled condition (AI_rev) and the Human-labeled condition (Human_rev), with 95% confidence intervals reported for AI_rev. As shown in Table 6, none of the five attitude items reached the conventional significance threshold (*p* < 0.05) under the AI labeled condition (AI_rev). Trust showed the highest confidence (*r* = 0.251, *p* = 0.053, 95% CI [−0.003, 0.475]) and Expectation the second highest (*r* = 0.238, *p* = 0.067, 95% CI [−0.016, 0.464]), both falling marginally short of the conventional threshold; crucially, both confidence intervals include zero, confirming the absence of reliable association. Replacement and Pressure showed negligible coefficients (*r*s = 0.048 and 0.002), and Vigilance, despite being the most widely endorsed attitude, yielded a weak coefficient that failed to reach statistical significance (*r* = 0.248, *p* = 0.056).

**Table 6:** Pearson correlations between AI-related attitudes and post-editing revision counts (*n* = 60).

Attitude item	r (AI_rev)	*p*	95% CI	Sig.	r (Human_rev)	*p*	Sig.
Vigilance	0.248	0.056	[−0.007, 0.472]	†	0.112	0.394	ns
Expectation	0.238	0.067	[−0.016, 0.464]	†	0.233	0.074	†
Trust	0.251	0.053	[−0.003, 0.475]	†	0.145	0.268	ns
Replacement	0.048	0.716	[−0.209, 0.298]	ns	0.187	0.152	ns
Pressure	0.002	0.988	[−0.252, 0.256]	ns	0.090	0.493	ns

r, represents Pearson correlation coefficient; *p*-values are two-tailed; †*p* < 0.10, ns, non-significant.

Comparison with Human_rev correlations, also shown in Table 6, reveals an important asymmetry. While Vigilance and Trust showed marginal trends in the AI-labeled condition, they yielded negligible and non-significant associations in the Human-labeled condition (*r*s = 0.112 and 0.145).

#### 4.3.3 Restricted variance and its implication for association magnitude

The weak associations observed across all five attitude items are in part attributable to the distributional characteristics documented in Table 5. As shown in Table 5, for the most highly endorsed items—Vigilance, Expectation, and Trust—the response range was substantially restricted (*SD*s ranging from 0.62 to 0.85), with the vast majority of participants concentrated at the upper end of the scale. When between-person variation in a belief item is this small, the statistical capacity to detect strong association with behavioral outcomes is correspondingly limited, regardless of whether a substantive relationship exists in principle. This distributional restriction is most acute for Vigilance: with 93.3% of responses concentrated at scale points 4 and 5, there is reduced variation across participants to reveal the full extent of differential associations with revision behavior. In contrast, Replacement and Pressure, which showed wider distributional spread, also showed the weakest associations with AI_rev (*r*s = 0.048 and 0.002), suggesting that variation in these belief dimensions was not behaviorally consequential in this sample.

In direct response to the third question, the extent to which variations in AI-related attitudes were associated with labeling-induced cognitive bias in post-editing behavior was statistically marginal. While the correlations did not reach the conventional *p* < 0.05 threshold, marginal trends emerged for Trust (*r* = 0.251) and Expectation (*r* = 0.238). The distributional restriction affecting the three most uniformly held beliefs further limits the extent to which individual-level belief variation could be detected as a correlate of behavioral bias in this sample.

## 5 Discussion

### 5.1 Source labeling as a dominant evaluative cue: evidence of schema-driven bias

The findings of first research question demonstrate that source labeling exerts a powerful and pervasive influence on both evaluative judgment and post-editing behavior. Drawing on the Source Credibility Framework (Hovland et al., 1953), this effect can be understood as a “source discounting” mechanism triggered by the AI label. Under mislabeling conditions, the label proved sufficient to reverse participants’ quality judgments entirely: the human-authored text, bearing an AI label, was judged as the lower-quality translation by 86.67% of experimental group participants, while the AI-generated text presented as human was rated as higher quality by 76.67% and incorrectly identified as the human translation by 93.33%. These inversions occurred not because the texts differed in linguistic quality but because the label determined the judgment (Dietvorst et al., 2015; Asscher and Glikson, 2023; Leuppert et al., 2025; Morosoli, 2025). At the quantitative level, mean total quality scores dropped by 1.32 points (d = 0.65) and revision counts rose by 17.8% on the basis of attribution alone—effects large enough to be consequential in professional evaluation and pedagogical assessment contexts. Maier et al. (2017) identify a parallel dynamic in their analysis of belief-consistency effects, whereby strong schema-identification leads individuals to evaluate content selectively. In the present context, the AI label functioned as a negative source cue that diminished perceived expertise and trustworthiness. Leuppert et al. (2025) further demonstrate that perceived AI authorship reduces credibility specifically on dimensions associated with interpretive judgment, independently of actual content quality. The systematic score suppression observed here may thus be understood not merely as an evaluative adjustment but as a behavioral manifestation of reduced credibility, whereby a form of skepticism leads to a “source-driven devaluation” of the message regardless of its objective merits (Racine, 2025).

The evaluative suppression extended beyond mean scores to the distributional structure of ratings. This shift indicates that the AI label does not merely suppress scores uniformly; rather, it restructures participants’ internal evaluative priorities. The AI label introduced greater evaluative volatility (*SD* = 1.48 vs. 1.35), suggesting that participants were less certain in their judgments when the source was attributed to a machine (Béchara et al., 2021; Yao et al., 2026). More consequentially, the 31.6-percentage-point collapse in Excellent-category ratings indicates that the label functioned as a “quality ceiling” that prevented the highest quality tier from being awarded regardless of the text’s intrinsic properties (Asscher and Glikson, 2023; Rossi, 2017; Zhou et al., 2023). This suggests that the AI label systematically lowered participants’ threshold for judging a text as requiring intervention, producing a distributional shift toward greater editing intensity. Szymczak’s (2016) research on translation quality assessment demonstrates that evaluator biases systematically affect both the assigned scores, and the implicit thresholds applied during evaluation, a finding that is directly paralleled here, where the operative threshold was set by the attributed source rather than the text. This ceiling effect aligns with Kameda’s (1986) early theoretical account of labeling effects, which argues that source-based expectancy dominates judgment most completely when message diagnosticity is low, with holistic quality rating providing precisely this condition due to its lack of a single objectively correct score.

At the behavioral level, the AI label produced a systematic downward shift in editorial tolerance: high-intensity revision rates doubled, minimal revision nearly disappeared. This pattern indicates that the AI label systematically lowered participants’ threshold for intervention, essentially redefining the “baseline of acceptability”. The convergence of this behavioral escalation with the attention self-rating data suggests a potential reallocation of attentional resources toward error-detection at the expense of stylistic refinement when a machine source is anticipated. This expectation-driven pattern aligns with the anchoring dynamic documented by Martikainen and Tiittula (2016) in machine translation post-editing research, where cognitive baselines established prior to textual engagement constrain what editors attend to and how they intervene. Revision count, as employed in the present study, functions as a behavioral indicator of cognitive effort in post-editing—a construct whose measurement has been approached through eye-tracking and pause analysis in prior research (O’Brien, 2006; Moorkens, 2018; Munková et al., 2021), and whose magnitude has been shown to vary with MT error type and perceived self-efficacy (Vardaro et al., 2019; Peng et al., 2024). The present findings extend this body of work by demonstrating that revision volume responds not only to objective text properties but to source attribution: the AI label activates an “increased scrutinization effort”(Ekinci, 2023), elevating revision counts as a defensive professional response to perceived machine untrustworthiness.

The qualitative data provide further triangulation for these patterns. The divergence in reflective vocabulary indicates that the AI label alone shaped the cognitive orientation brought to the task, reducing the diagnostic weight of stylistic naturalness while over-emphasizing semantic accuracy. Experimental group participants’ dominance of error-detection vocabulary (*cuo wu*, *luo ji*) versus the control group’s holistic terms (*sheng ying*, *wen hua*) confirms that surface-level stylistic naturalness is the primary target of AI-labeled-induced negative expectations. This pattern is consistent with the confirmation bias dynamic described by Ali (2025) in translation contexts, whereby initial source expectations redirect selective attention toward evidence that confirms the preconception, and with Wang’s (2020) cognitive psychology framework, which identifies selective attention to source-salient features as a primary mechanism through which evaluative biases emerge and persist. In response to Q18, terms such as *ke ban* [stereotyped] (*n* = 2) and *yin xiang* [impression] (*n* = 2) appeared among mislabeled participants alongside the dominant *ying xiang* [influence] terms. The emergence of these descriptors suggests that the bias persisted even among those with partial metacognitive awareness of its presence. Ultimately, the AI label functions as a “heuristic shortcut” that pre-sets the evaluator’s gaze toward localized faults, reinforcing a lower-order evaluative schema even when the evaluator possesses the professional capacity for higher-order engagement.

### 5.2 Differential susceptibility of evaluative criteria and the restructuring of evaluative priorities

The evidence gathered for the second research question illustrates that the AI label did not suppress translation quality judgments uniformly but acted selectively across evaluative dimensions, with consequences that extend beyond score reduction to the internal structure of how quality was assessed. The data establishes a clear hierarchy of susceptibility: while all scores declined, the magnitude of this effect was markedly tiered. Fluency was most severely affected (*d* = −0.94), followed by Fidelity (*d* = −0.56), with Completeness proving most resilient (*d* = −0.49). This gradient is not arbitrary. Fluency indexes stylistic naturalness, idiomatic expression, and register appropriateness: the properties most directly associated with what human translation is understood to do irreducibly well. When an AI label is applied, the schema it activates is most specifically a schema of stylistic deficiency, making Fluency the dimension most exposed to schema-congruent negative projection (Asscher and Glikson, 2023; Ilić and Damnjanović, 2021; Mena, 2025). Fidelity and Completeness, being more structurally verifiable (i.e., whether the translation accurately and completely renders the source), are less amenable to this kind of expectancy-driven penalty. This ordering maps precisely onto Kameda’s (1986) theoretical account: labeling effects are strongest when message diagnosticity is low, and Fluency, as the criterion with no single objectively correct benchmark, provides exactly the low-diagnosticity conditions under which prior expectancy dominates judgment most completely.

The weighting shift documented in the correlation analysis adds a structurally distinct dimension to this picture. Under human labeling, Fluency was the primary criterion in participants’ holistic quality judgment (*r* = 0.791); under AI labeling, its diagnostic weight declined notably (*r* = 0.674, *Δ* = −0.117), and Fidelity displaced it as the leading correlate of total quality (*r* = 0.740). The label thus did not merely lower scores—it reorganized what participants were implicitly using as their principal standard of quality, shifting the evaluative question from stylistic appreciation to accuracy verification. Munday’s (2012) analysis of evaluation in translation identifies how cognitive biases create systematic patterns at critical decision points where quality standards are applied; the present weighting shift suggests that source attribution functions as precisely such a decision point—one that precedes textual engagement and restructures the evaluative frame before any criterion is consciously applied. Litwin’s (2025) finding that anchoring effects constrain subsequent evaluative choices by establishing an initial cognitive reference point supports the same interpretation at the structural level. This structural reorganization emphasizes the necessity of integrating artificial and human intelligence more effectively in professional workflows (Herbig et al., 2019).

The simultaneous direct penalization and reduced weighting of Fluency reflects a dual-level process that merits attention. Evaluators both penalized stylistic imperfections more heavily under the AI label and accorded those imperfections less diagnostic weight in holistic judgment. These two effects are not contradictory: when stylistic awkwardness is anticipated as a category-typical feature of machine output, observing it confirms the schema rather than differentiating the text from expectation. The penalty is applied, but its signal value for overall quality assessment is diminished. Szymczak’s (2016) research shows that evaluator biases affect not only assigned scores but the implicit prioritization of criteria during quality assessment; the present findings extend this insight by demonstrating that a single source cue can produce opposite-direction effects on the same criterion simultaneously, manifesting as harsher direct penalization alongside reduced diagnostic significance. This represents a dynamic that a simple score-suppression model cannot account for.

### 5.3 AI-related attitudes as professional norms rather than behavioral predictors

Given the specific distributional properties of the attitude items, the non-significant correlation between belief strength and AI-labeled revision counts reported in RQ3 transcends a mere null finding. Instead, it offers a theoretically informative perspective on the underlying cognitive processes. The three most highly endorsed items—Vigilance, Expectation, and Trust—were endorsed at rates of 93.3, 83.3, and 68.3% respectively, with standard deviations between 0.62 and 0.85. Statistical association between belief strength and behavioral outcomes requires sufficient between-person variation in the predictor; where a belief is held at near-universal intensity within a professional cohort, individual differences in belief strength cease to function as differentiating variables (Cohen, 2003; Maier et al., 2017). Therefore, these findings suggest that within this specific professional cohort, AI-related attitudes do not function as significant linear predictors of labeling-induced bias. Instead, the observed bias appears to be a generalized cognitive response to the ‘AI’ label itself rather than being contingent upon individual variations in trust or anxiety, particularly within the restricted range of beliefs captured in this sample (Ilić and Damnjanović, 2021; Morosoli, 2025).

The null finding does not indicate that these beliefs are irrelevant to labeling bias—it indicates that within this sample they have become so uniformly distributed that they operate as shared professional background conditions rather than as variable individual predictors. Komeili et al. (2019) argue that metacognitive awareness of cognitive biases and heightened scrutiny toward AI-attributed content become ingrained features of professional cognition as translation training progresses, shifting from variable personal traits to normative professional stances. Prassl’s (2017) longitudinal findings similarly show that translators develop deeply entrenched cognitive routines that are stable across practitioners rather than differentiating between them. The present sample, comprising MA-level students with substantial formal training, appears to have reached precisely this stage: AI vigilance has crystallized into a shared professional norm. Consequently, these findings represent a case of statistical nullity that yields substantial conceptual significance. The results suggest that the labeling effect functions as a universal cognitive schema rather than an idiosyncratic reaction; once a belief reaches such near-universal intensity, it transitions from an individual variable to an environmental constant, thereby precluding the detection of linear associations within this professional cohort.

The Vigilance correlation trend sharpens this interpretation. Vigilance yielded a marginal positive trend with AI_rev (*r* = 0.248, *p* = 0.056) but was negligible with Human_rev (*r* = 0.112, *p* = 0.394). Given the post-experimental nature of the questionnaire, this pattern suggests a retrospective alignment between belief and behavior. Participants who revised more extensively under the AI label were more likely to rationalize their effort as a manifestation of professional vigilance. This partially aligns with Lehka-Paul’s (2020) research, which demonstrates that individual differences in editing intensity reflect relatively stable decisional styles. However, the current study adds a layer of “cognitive labeling” to this trait-based view: while participants may possess a stable trait of high editing effort, they selectively attribute this effort to “AI vigilance” only when an AI label is present (Bo and Yuan-yi, 2015; Borg, 2017). The most widely endorsed belief in the dataset thus did not produce proactive AI-specific behavioral differentiation; instead, it functioned as a post-hoc justification for the labeling effect already in play. This pattern underscores a decoupling between explicit beliefs and implicit task behavior, suggesting that even when participants perceive themselves as more vigilant, their behavioral response remains governed by an ingrained, instinctive labeling-induced bias that operates independently of their intellectual stance.

Integrating the evidence from both group-level score analysis and individual-level belief correlations, these findings indicate that the operative mechanism is the contextual cue, namely the label, rather than the strength of any individual’s prior belief. This indicates that the situational strength of the source cue—the AI label—overrides individual psychological predispositions (Ross and Nisbett, 2011). The label acts as a powerful environmental trigger that activates a standardized defensive editing mode, demonstrating that the bias is so deeply internalized through professionalization that it functions as a collective behavioral standard rather than a contingent individual response. Greitemeyer et al. (2009) demonstrate that biased assimilation operates as a function of source position rather than recipient characteristics, supporting exactly this conclusion. This has a direct practical implication: if the mechanism is situational rather than dispositional, interventions targeting individual attitude change are unlikely to be sufficient. Given the current findings, workflow-level redesigns, such as delayed source disclosure, blind post-editing protocols (O’Brien, 2007), and rubric-guided assessment that separates criterion-level judgment from holistic rating, may offer more immediate and predictable interventions than programs aimed solely at reshaping professional beliefs. The latter, as observed in this study, may have already become too uniformly distributed or decoupled from action to function as reliable behavioral differentiators in high-pressure task environments.

## 6 Conclusion

This study demonstrates that perceived source identity, rather than objective translation quality, constitutes a systematic driver of evaluative judgment and post-editing behavior among trained translation students. When source labels diverged from actual text origin, quality judgments inverted, revision intensity escalated, and the implicit criteria through which quality was assessed were restructured, with stylistic naturalness giving way to semantic accuracy as the dominant evaluative standard. That these effects operated uniformly regardless of individual belief strength further indicates that labeling-induced bias in this context is primarily a property of the evaluation situation rather than of the individual evaluator, pointing to workflow design rather than just attitudinal change as the more tractable site of intervention. By situating these findings at the intersection of translation studies and cognitive psychology, the study provides empirical grounding for what may be characterized as a subtle but consequential form of source-identity bias in professional post-editing contexts and offers a foundation for developing pedagogical approaches that cultivate critical post-editing literacy and more calibrated attitudes toward AI-mediated translation.

Regarding the study’s limitations, the findings are based on a focused sample from a single MTI program and a limited set of translation stimuli. While providing a controlled environment for observing labeling effects, this specific scope may constrain their generalizability across broader professional contexts, diverse language pairs, and varied text types. To build on this foundation, future research should employ more diverse participant profiles and varied materials to test the robustness of the dimensional susceptibility hierarchy documented here, examine how labeling effects interact with professional experience and occupational identity, and investigate whether workflow-level interventions can effectively disrupt the schema-activation pathway through which source attribution currently operates.

## Data Availability

The raw data supporting the conclusions of this article will be made available by the authors, without undue reservation.

## Appendix A: post-task questionnaire survey

Instructions: Please complete this survey based on your genuine feelings. Your feedback is greatly appreciated. Thank you!

1. Gender:

○ Male○ Female

2. Rage of age:

○ 18~25○26~30○31~40

3. Year of master study:

○ Grade 1○ Grade 2○ Grade 3

4. English Proficiency (CET-6/TEM-8/IELTS/TOEFL) __________
  5. Translation Experience ____________
  6. When revising what you believed to be the “translated text”, to what extent did you attend to the following aspects? (1 = Not at all attentive, 5 = Extremely attentive)

Correction of linguistic errors   ① ② ③ ④ ⑤
Fluency of expression  ① ② ③ ④ ⑤
Cultural appropriateness   ① ② ③ ④ ⑤
Consistency (terminology, expression, or style)① ② ③ ④ ⑤

7. I believe Al translation quality is steadily improving. (1 = Strongly Disagree, 5 = Strongly Agree)

① ② ③ ④ ⑤

8. I tend to trust human translators' judgment more than Al systems’. (1 = Strongly Disagree, 5 = Strongly Agree)

① ② ③ ④ ⑤

9. When I believe that the translation is produced by AI, I check for errors more carefully. (1 = Strongly Disagree, 5 = Strongly Agree)

① ② ③ ④ ⑤

10. I believe that AI translation can replace part of human translators’ work in the future. (1 = Strongly Disagree, 5 = Strongly Agree)

① ② ③ ④ ⑤

11. I feel greater pressure or doubt when revising AI-generated translations. (1 = Strongly Disagree, 5 = Strongly Agree)

① ② ③ ④ ⑤

12. When comparing the two translations, which one do you consider to be of higher overall quality?

○ Text A○ Text B○ About the same○ Not sure

13. Which translation do you believe was generated by AI?

○ Text A○ Text B○ Not Sure

14. Which translation did you find easier to revise?

○ Text A○ Text B○ About the same○ Not sure

15. Which translation did you feel more confident revising?

○ Text A○ Text B○ About the same○ Not sure

16. Which translation, in your opinion, better reflects a “human” translation style?

○ Text A○ Text B○ About the same○ Not sure

17. How did your experience differ when revising the “AI-labeled” translation versus
  the “Human-labeled” one?

__________________________________________________________________________

18. Do you believe that the “AI” or “Human” labels influenced your judgment or editing behavior? Please explain why.

___________________________________________________________________________
